# Six decades of trends in BMI, thinness, overweight, and obesity among 210,927 Swedish children in different socioeconomic contexts

**DOI:** 10.1186/s12889-026-29521-4

**Published:** 2026-09-18

**Authors:** Maria Bygdell, Klara Hemmed, Jari Martikainen, Carl Bonander, Lovisa Sjögren, Claes Ohlsson, Jenny M Kindblom

**Affiliations:** 1 Department of Internal Medicine and Clinical Nutrition, Institute of Medicine, Sahlgrenska Academy, University of Gothenburg, Vita stråket 11, Gothenburg, 413 45 Sweden; 2 Unit of Clinical Pharmacology, Department of Pharmaceuticals, Sahlgrenska University Hospital, Region Västra Götaland, Gothenburg, Sweden; 3 Research and Development Primary Health Care and Kungshöjd Pediatric Clinic, Region Västra Götaland, Gothenburg, Sweden; 4 Bioinformatics and Data Centre, Sahlgrenska Academy, University of Gothenburg, Gothenburg, Sweden; 5 School of Public Health and Community Medicine, Sahlgrenska Academy, University of Gothenburg, Gothenburg, Sweden; 6 Karlstad Business School, Karlstad University, Karlstad, Sweden; 7 Department of Pediatrics, Institute of Clinical Sciences, Sahlgrenska Academy, University of Gothenburg, Gothenburg, Sweden; 8 MOVMED, Carlanderska Hospital, Gothenburg, Sweden

**Keywords:** Childhood obesity, Trends, Socioeconomic contexts

## Abstract

**Background:**

Childhood obesity and low socioeconomic status increase the risk for adverse health outcomes. Societal changes may have altered the association between parental socioeconomic factors and childhood obesity over time. Our aim was to evaluate the trends for BMI in girls and boys 1952–2017 and the importance of socioeconomic factors for the BMI trend 2002–2017.

**Methods:**

We used the population-based BMI Epidemiology Study cohort with information on BMI for seven-year-old children between 1952 and 2017 in Gothenburg, Sweden. We included in total 210,927 children and assessed the overall trend as well as in different socioeconomic strata. Data on parental country of birth, educational level, and income were available for a subpopulation between 2002 and 2017 (*n* = 65,556).

**Results:**

Childhood BMI trends showed four distinct phases: a modest increase 1952–1973, a steep increase 1974–2001, a brief period of decline 2002–2006, followed by diverging trends 2008–2017 that differed by parental socioeconomic strata. In the latter period, children with Swedish-born parents, high educational level, and high income displayed a stabilising trend, whereas children with parents born outside Sweden, with low educational level, and low income showed increasing BMI trends. Parental low educational level had the most pronounced increasing BMI trend in their children, regardless of income and country of birth.

**Conclusions:**

After increasing BMI and a brief period of decline, we found widening socioeconomic disparities in childhood BMI 2008–2017, with the most pronounced increases among children whose parents had low educational level. These findings highlight the need for targeted interventions.

**Supplementary Information:**

The online version contains supplementary material available at https://doi.org/10.1186/s12889-026-29521-4.

## Introduction

The global prevalence of childhood overweight and obesity remains alarmingly high [1]. A high BMI in childhood increases the risk of overweight and obesity in adulthood [2, 3]. Additionally, a high BMI is also associated with a heightened risk of numerous adult-onset diseases, including cardiovascular disease, several types of cancers, type 2 diabetes, obstructive sleep apnoea, dyslipidaemia, metabolic dysfunction-associated steatotic liver disease, and orthopaedic complications [4–7]. Furthermore, children with obesity face a threefold increased risk of premature mortality in early adulthood compared to peers from the general population [6]. Beyond physical health consequences, childhood obesity is linked to increased psychological distress, including anxiety, stress, and a higher likelihood of being bullied [8], and even increased risk for suicide [6]. Academic performance may also be negatively affected; evidence suggests that children with obesity achieve lower educational outcomes, even after adjusting for socioeconomic status [9].

Over the past several decades, childhood BMI and the prevalence of overweight and obesity have risen globally [1, 2]. Data from some countries indicate a potential plateau or stabilisation from the 1990s and onwards in these increasing trends [10] but whether this shift is consistent across different socioeconomic strata remains unclear. Findings from the United Kingdom (UK) suggest widening socioeconomic disparity in childhood BMI among cohorts born after 2001 compared to earlier generations based on the fathers’ occupation as socioeconomic factor, using data from around 57,000 7 to 15 years-old individuals [11]. A previous study from Sweden showed that there were increased inequalities in obesity prevalence among 7–9-year-old children between 2008 and 2013 using area-level educational level as the socioeconomic variable [12]. Another study showed that the prevalence of obesity among 10-year-old children was more prevalent in socioeconomic disadvantaged areas in Sweden during 1999–2003 [13]. However, less is known about which family-level socioeconomic factors are most strongly associated with these secular changes in BMI, limiting opportunities to target interventions effectively.

We hypothesised that BMI trends among seven-year-old children in Sweden differ according to parental socioeconomic status and demographic factors such as country of birth, educational level, and income. Our aim was to evaluate the trends for BMI, including thinness, overweight, and obesity, in seven-year-old girls and boys 1952–2017 and the importance of socioeconomic factors for the BMI trend 2002–2017.

## Methods

### Study design and data sources

This study was conducted using the population-based BMI Epidemiology Study (BEST) cohort that includes individuals in the Gothenburg municipality, Sweden. During the last 100 years, all children in Sweden have attended regular health visits at child and school health services (> 98.5% of all pupils attended school health services from calendar year 1952) [14]. Height and weight measurements were recorded in healthcare records that followed the child throughout school and were stored in a central archive in Gothenburg after the child finished school. We collected data on height and weight from these healthcare records and linked them with data from the national registers in Sweden, using the personal identity number (PIN). All citizens in Sweden are assigned a PIN at birth or immigration. Using information from the Multigeneration Register, we were able to link all children with their parents and retrieve information on parental country of birth, educational level, income, and age at the birth of the child from the Longitudinal Integration Database for Health Insurance and Labor Market Studies (LISA database). This study was conducted in accordance with the Declaration of Helsinki and has been approved by the ethics committee at Gothenburg University (Dnr 013 − 10 with several amendments).

### Study population

The inclusion criterion for this study was to have at least one measurement of height and weight on the same date between six and eight years of age for the calculation of childhood BMI at the age of seven. We included all seven-year-old children who fulfilled the inclusion criterion in 1952–1975, 1978, 1983, 1988, 1993, 1998–2006, and 2008–2017, in total 210,927 individuals. Due to missing data, not all years between 1952 and 2017 could be included.

### Exposure and outcome

We evaluated the trend in childhood BMI using the year the included children turned seven as exposure, and childhood BMI as a continuous variable, as well as BMI categories, as outcomes. BMI was calculated by dividing body weight (in kg) with height (in m^2^) using all paired height and weight measurements between six and eight years of age. The interval six to eight years was selected to represent the childhood period, after infancy and before puberty. As BMI is age-dependent during development, we performed age adjustment within the age interval. A linear model was fitted using all BMI measurements between six and eight years of age, separately by gender, with BMI as dependent variable and age as independent variable. The regression coefficient for age represented the average annual change in BMI between six and eight years within each gender. This coefficient was then used to standardize each child’s observed BMI to age seven by accounting for the average expected change in BMI between the child’s age at measurement and seven years. BMI was also categorized into thinness, normal weight, overweight, and obesity using the age- and sex-specific cut-offs from the International Obesity Task Force (IOTF) [15].

### Covariates

Information on parental socioeconomic status was available for children with measurements from 2002 and onwards. Parental country of birth was categorised as Sweden (both parents born in Sweden) or not Sweden (one or both parents not born in Sweden). Parental educational level was categorised as low (12 years or shorter education for both parents at the age of seven for the child), high (longer than 12 years of education for one or both parent at the age of seven for the child), or unknown. Parental income was calculated as the sum of the parents’ disposable income and categorised according to the median each year; low (below median), high (above median), or unknown. The age of each parent at childbirth, respectively, was used as continuous variables. The full model with year as exposure and continuous or categorized BMI as outcome also included gender, parental country of birth, parental educational level, parental income, and parents’ age at childbirth as covariates. In a sub analysis, parental country of birth was split into three groups; both parents born in Sweden, one parent born in Sweden, and no parent born in Sweden.

### Statistical analyses

Descriptive statistics for childhood BMI and BMI categories were calculated according to gender and year. Childhood BMI is presented as mean (SD or SE) and BMI categories are presented as number (%). The overall trend of childhood BMI and BMI categories over measurement years were analysed using linear regression models and logistic regression models, respectively. The distribution of childhood BMI according to years was described using sex-specific BMI percentiles 5th, 25th, 50th, 75th, 85th, 95th, and 99th. Modelling of a non-linear trend was performed using splines, and differential trends were evaluated by including strata-time interactions into the model. A p-value < 0.05 for the interaction terms were considered a significant interaction. Further analysis of non-linearity was performed using a piecewise linear regression model where the number and placement of the breakpoints were determined by the model yielding the lowest Akaike Information Criterion. Further analysis of the socioeconomic factors’ contribution of differential trends was performed using sub-analyses. Correlation between the socioeconomic variables were evaluated by Pearson’s correlation coefficient in a cross-sectional analysis. For all statistical analyses, R v.4.5.0 (standard packages, quantreg, and segmented) was used.

## Results

We included 210,927 children with childhood BMI at the age of seven between 1952 and 2017 (Supplemental Table S1 and Figure S1). The mean childhood BMI was 15.75 (SD: 1.73) kg/m^2^ for the entire cohort. The prevalence of the different BMI categories is presented in Supplemental Table S2.

### Trends in childhood BMI between 1952 and 2017

**Fig. 1:**
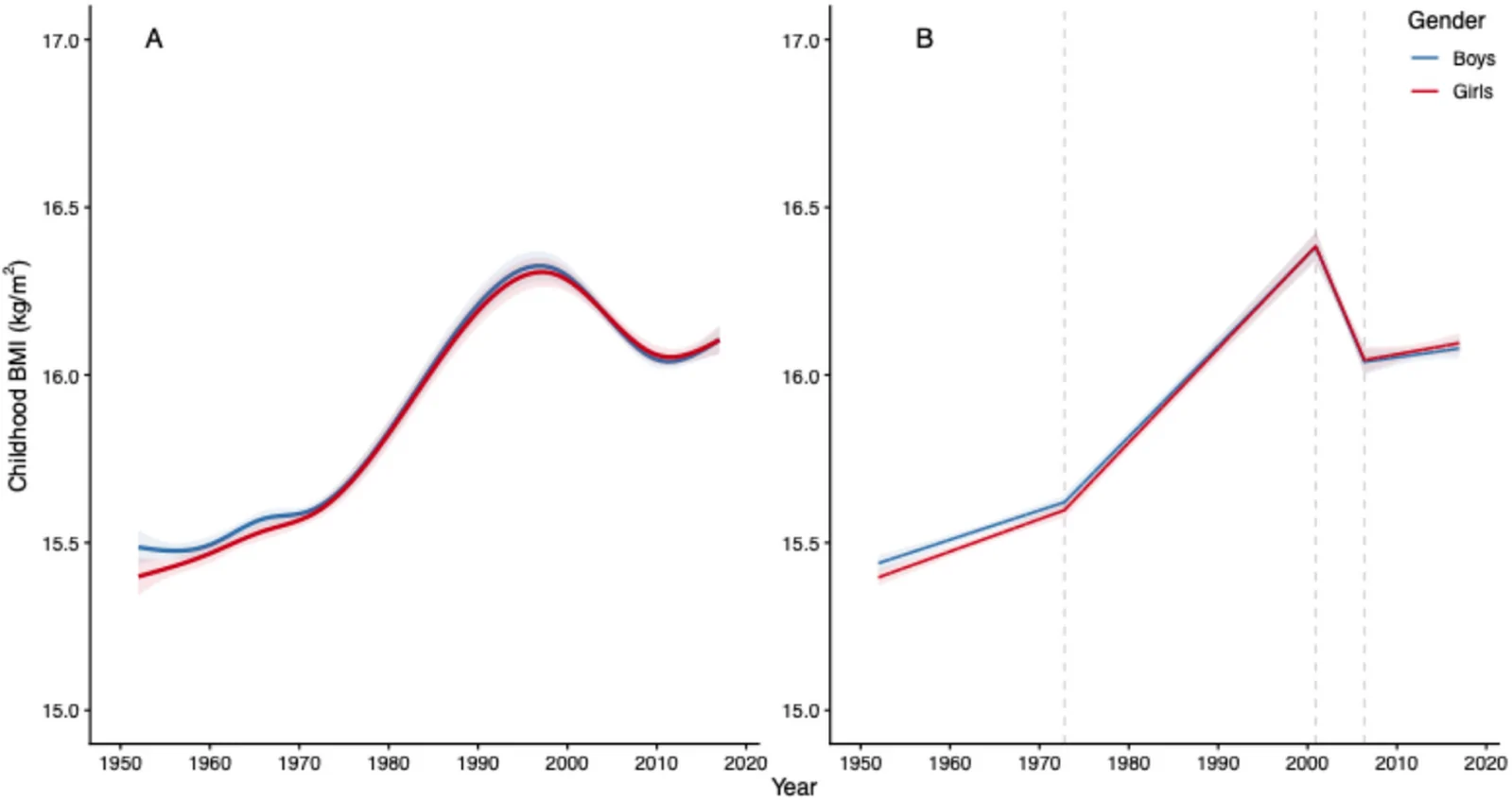
BMI at the age of seven measured between 1952 and 2017 for 210,927 children in schools in Gothenburg municipality, Sweden. **A** Raw BMI data fitted with splines and their 95% confidence intervals. **B** Regression lines and their 95% confidence intervals from a piecewise linear regression model with three breakpoints indicated (dotted lines)

Modelling the association between year and childhood BMI using splines indicated non-linearity (Fig. 1a ). A piecewise linear regression model revealed three breakpoints, dividing the study period into four phases with different estimates for the association (phase 1 = 1952–1973, phase 2 = 1974–2001, phase 3 = 2002–2006, and phase 4 = 2008–2017) (Fig. 1b). There was a statistically significant interaction between year and gender in the association with childhood BMI (*p* < 0.01). Therefore, separate analyses for the two genders were performed. We observed a modest increase 1952–1973 (Table 1). The steepest increase in childhood BMI was observed in phase 2 1974–2001 (Beta: 0.14 [95%CI: 0.12;0.15] kg/m^2^ per five years for boys and 0.14 [0.12;0.15] for girls). There was a pronounced decrease in phase 3 2002–2006 (boys: -0.37 [-0.49;-0.24] and girls: -0.37 [-0.50;-0.23]), and diverging trends 2008–2017 (Table 1, Table 2).

**Table 1:** BMI at the age of seven between 1952 and 2017 for 210,927 children in schools in Gothenburg municipality, Sweden. Beta values (kg/m^2^) per five years from a piecewise linear regression model

	Boys		Girls	
	Beta (95%CI) kg/m^2^ per five years	*p*-value	Beta (95%CI) kg/m^2^ per five years	*p*-value
Phase 1 (1952–1973)	0.04 (0.03;0.05)	< 0.001	0.04 (0.03;0.05)	< 0.001
Phase 2 (1974–2001)	0.14 (0.12;0.15)	< 0.001	0.14 (0.12;0.15)	< 0.001
Phase 3 (2002–2006)	-0.37 (-0.49;-0.24)	< 0.001	-0.37 (-0.50;-0.23)	< 0.001
Phase 4 (2008–2017)	0.02 (-0.02;0.05)	> 0.05	0.02 (-0.02;0.06)	> 0.05

*BMI *Body Mass Index, *CI *Confidence interval

**Table 2:** Overweight and obesity at the age of seven between 1952 and 2017 for children in schools in Gothenburg municipality, Sweden. OR per five years from separate stratified logistic regression models. Overweight and obesity compared to not overweight and not obesity, respectively

	Boys		Girls	
	OR (95%CI) per five years	*p*-value	OR (95%CI) per five years	*p*-value
Overweight				
Phase 1 (1952–1973)	1.11 (1.08;1.15)	< 0.001	1.11 (1.08;1.14)	< 0.001
Phase 2 (1974–2001)	1.22 (1.19;1.26)	< 0.001	1.16 (1.14;1.19)	< 0.001
Phase 3 (2002–2006)	0.68 (0.53;0.86)	< 0.01	0.79 (0.63;0.99)	< 0.05
Phase 4 (2008–2017)	1.07 (1.00;1.15)	> 0.05	1.09 (1.02;1.16)	< 0.05
**Obesity**				
Phase 1 (1952–1973)	1.14 (1.06;1.24)	< 0.001	1.18 (1.11;1.26)	< 0.001
Phase 2 (1974–2001)	1.48 (1.39;1.57)	< 0.001	1.33 (1.27;1.39)	< 0.001
Phase 3 (2002–2006)	0.47 (0.31;0.71)	< 0.001	0.74 (0.52;1.06)	> 0.05
Phase 4 (2008–2017)	1.16 (1.03;1.30)	< 0.05	1.04 (0.93;1.17)	> 0.05

*CI *Confidence interval, *OR *Odds ratio

The increasing BMI over time was observed across the 85th, 95th, and 99th percentiles of the childhood BMI distribution (Supplemental Figure S3).

### Trends in thinness, overweight, and obesity between 1952 and 2017

There was a statistically significant interaction between year and gender in the association with BMI categories (*p* < 0.01). Therefore, separate analyses for the two genders were performed. Logistic regressions between year and BMI categories in the four separate phases revealed that for every five years in phase 2 (1974–2001), there was an increase in the odds for overweight (boys: OR 1.22 [95%CI 1.19;1.26] and girls: 1.16 [1.14;1.19]) and obesity (boys: 1.48 [1.39;1.57] and girls: 1.33 [1.27;1.39]), and in phase 3 (2002–2006), there was a decrease in the odds for overweight (boys: 0.68 [0.53;0.86] and girls: 0.79 [0.63;0.99]) and obesity for boys (0.47 [0.31;0.71]) but not girls (Table 2). In contrast, there was a decrease in thinness in boys (0.98 [0.98;0.99]) and girls (0.96 [0.96;0.97]) over the entire period 1952–2017 (Supplemental Figure S4).

### Trends in childhood BMI in socioeconomic strata between 2002 and 2017

**Fig. 2:**
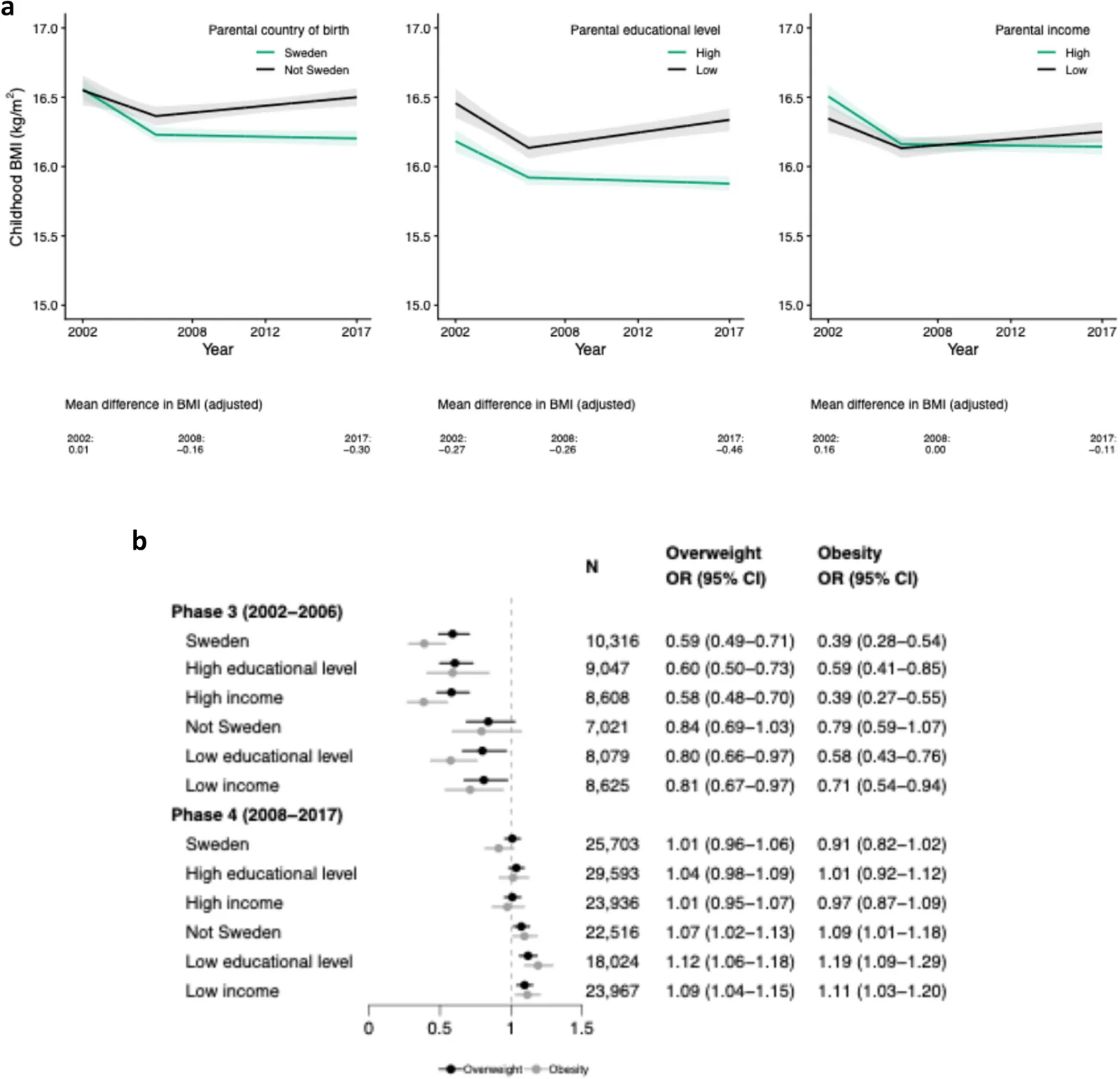
**a**. The trend in BMI at the age of seven between 2002 and 2017 for 63,888 children in schools in Gothenburg municipality, Sweden, by **A** parental country of birth, **B** parental educational level, and **C** parental income. Regression lines and their 95% confidence intervals from a piecewise linear regression model with one breakpoint and including gender, parents’ age at childbirth, and the socioeconomic variables not stratified on. **b** The odds for overweight and obesity at the age of seven between 2002 and 2017 for 63,888 children in schools in Gothenburg municipality, Sweden, by parental country of birth, parental educational level, and parental income. Odds ratio and their 95% confidence intervals per five years from logistic regression models in the two phases and including gender, parents’ age at childbirth, and the socioeconomic variables not stratified on. Overweight and obesity compared to not overweight and not obesity, respectively.

Next, we evaluated the potential effect modification of the socioeconomic factors in the association between year and childhood BMI in phase 3 and 4 (2002–2017). For cross-sectional correlations, descriptives, and associations of the socioeconomic factors, please see Supplemental Table S3, S4, and Figure S5. In a model evaluating the BMI trend during these years, interaction terms between year and parental country of birth (*p* < 0.001), income (*p* < 0.05), and parental educational level (*p* < 0.05), respectively, but not between year and gender (*p* > 0.05), were significant in the full model. This indicated effect modification by the socioeconomic variables for the observed association. In separate multivariable piecewise linear regression analyses stratified for each socioeconomic variable and adjusted for the other two socioeconomic variables in addition to gender and parents’ age at childbirth, there was a decreasing trend of childhood BMI in phase 3 (2002–2006) in all strata, although with different beta values. However, in phase 4 (2008–2017), children with parents not born in Sweden, low educational level, and low income showed an increasing trend (Table 3, Fig. 2a), while the trend for children with parents born in Sweden, high educational level, and high income stabilised. In a sub analysis, children with no parent born in Sweden displayed an increasing trend in BMI whereas children in the groups with one or two parents born in Sweden showed a stabilisation (Supplemental Figure S6). An intersectional analysis revealed that children with parents with low educational level, regardless of income or country of birth, had the most pronounced increasing trend from 2008 (Table 4).

**Table 3:** BMI at the age of seven between 2002 and 2017 for children in schools in Gothenburg municipality, Sweden. Beta values (kg/m^2^) per five years from separate stratified linear regression models adjusted for gender, parents’ age at childbirth, and the socioeconomic variables not stratified on

	Beta (95%CI) kg/m^2^ per five years	*n*
Parental country of birth		
Sweden		35,702
Phase 3 (2002–2006)	-0.41 (-0.51;-0.31)	
Phase 4 (2008–2017)	-0.01 (-0.04;0.02)	
Not Sweden*		28,186
Phase 3 (2002–2006)	-0.23 (-0.39;-0.07)	
Phase 4 (2008–2017)	0.06 (0.02;0.10)	
Parental educational level		
High		38,168
Phase 3 (2002–2006)	-0.33 (-0.44;-0.22)	
Phase 4 (2008–2017)	-0.02 (-0.05;0.01)	
Low		25,191
Phase 3 (2002–2006)	-0.40 (-0.55;-0.25)	
Phase 4 (2008–2017)	0.09 (0.05;0.14)	
Parental income		
High		32,533
Phase 3 (2002–2006)	-0.43 (-0.54;-0.32)	
Phase 4 (2008–2017)	-0.01 (-0.04;0.02)	
Low		31,063
Phase 3 (2002–2006)	-0.27 (-0.41;-0.12)	
Phase 4 (2008–2017)	0.05 (0.01;0.09)	

*BMI *Body mass index, *CI *Confidence interval

*One parent not Sweden: Beta: 0.01 (95%CI: -0.02;0.04), *n* = 9018

Both parents not Sweden: Beta: 0.08 (95%CI: 0.03;0.14), *n* = 19,168

**Table 4:** Intersectional analysis of the trend in BMI at the age of seven between 2008 and 2017 for 46,474 children in schools in Gothenburg municipality, Sweden. Beta values (kg/m^2^) per five years from separate stratified linear regression models adjusted for gender

Group (Parental country of birth, income, educational level)	Beta (95% CI) kg/m^2^ per five years	*p*-value	*n*
Not Sweden, High, Low	0.25 (0.06;0.45)	< 0.05	1565
Not Sweden, Low, Low	0.14 (0.05;0.23)	< 0.01	8850
Sweden, Low, Low	0.09 (-0.03;0.22)	> 0.05	3155
Not Sweden, Low, High	0.05 (-0.05;0.15)	> 0.05	6373
Sweden, High, Low	0.01 (-0.09;0.11)	> 0.05	3701
Sweden, High, High	-0.03 (-0.07;0.02)	> 0.05	14,386
Sweden, Low, High	-0.08 (-0.16;0.01)	> 0.05	4170
Not Sweden, High, High	-0.10 (-0.20;-0.004)	< 0.05	4274

### Trends in thinness, overweight, and obesity in socioeconomic strata between 2002 and 2017

Next, we evaluated interactions between year and the different socioeconomic factors in logistic regression models including gender and parents’ age at childbirth for thinness, overweight, and obesity. There were no significant interactions for thinness, while effect modification was indicated by significant interactions between year and parental country of birth, educational level, and income, for overweight (*p* < 0.05) as well as parental country of birth (*p* < 0.001) and income (*p* < 0.05) for obesity. In separate multivariable logistic regression analyses stratified for each socioeconomic variable and adjusted for the other two socioeconomic variables in addition to gender and parents’ age at childbirth, there was decreased risk for overweight and obesity in phase 3 (2002–2006) for children with parents born in Sweden, with high educational level, and with high income. Importantly, in phase 4 (2008–2017) we observed an increased risk for overweight and obesity for children with parents not born in Sweden, with low educational level, and with low income (Fig.2b, Supplemental Figure S6). In a sub analysis, children with no parent born in Sweden displayed an increased risk for overweight and obesity whereas children in the groups with one or two parents born in Sweden showed a stabilisation (Supplemental Figure S7).

## Discussion

In this extensive population-based cohort study, we analysed long-term trends in BMI and BMI categories; thinness, overweight, and obesity among seven-year-old girls and boys in Gothenburg, Sweden, spanning the years 1952 to 2017. We observed a substantial rise in mean BMI, as well as in the prevalence of overweight and obesity up to 2001, followed by a subsequent decline. However, the trend from 2008 and onwards varied substantially across socioeconomic groups; children with parents not born in Sweden, with low educational level, and low income showed an increasing trend, while BMI for children with parents born in Sweden, with high educational level, and with high income stabilised after the previous decrease. Notably, children to parents with low educational level, regardless of income or country of birth, had the most pronounced upward trend from 2008.

Compilations of extensive data sets have revealed a significant rise in childhood obesity across most regions of the world over the past 30 years [1, 16]. Between 1990 and 2021, the combined prevalence of overweight and obesity doubled, while obesity rates alone tripled [16]. In recent years, however, evidence suggests that this upward trend may be levelling off in some countries, with signs of stabilisation, albeit at persistently high levels [10]. In line with these global patterns, our study observed a marked increase in BMI, overweight, and obesity among seven-year-old girls and boys, beginning 1974 and peaking 2001. The highest mean BMI values and the greatest prevalence of overweight and obesity were recorded in the early 2000s, corroborating findings from our previous study focused on boys [17]. The present study extends to include both girls and boys, a larger time span, and a detailed analysis of the socioeconomic components that might be of importance for BMI in children. We observed that the BMI peak was followed by a decline in the early years of the new millennium. The underlying causes of the decline remain unclear, but based on results from trials on prevention demonstrating positive effects on BMI development [18–20], it has been suggested that heightened awareness of the health risks associated with childhood obesity may have contributed to this positive shift. Moreover, the Swedish Public Health Agency, through its ongoing surveillance of health and health related behaviours among the Swedish population, reports that the consumption of sweetened beverages has declined among Swedish school aged children. This trend is documented in their national monitoring of dietary habits [21] and may have contributed to the trend observed in the present study.

In the UK, longitudinal data suggest that socioeconomic disparities in BMI have widened substantially from the 1940s to the 2000s [11]. The study, using the fathers’ occupation as proxy for socioeconomic status, did not evaluate the impact of separate socioeconomic factors. Importantly, a trend towards stronger tracking in groups with higher BMI levels and low socioeconomic status has been observed [22, 23]. Evidence further indicates that both individual-level [11] and neighbourhood-level [24] socioeconomic status are linked to obesity rates, suggesting that both intra-familial and broader societal mechanisms might contribute. Regarding specific socioeconomic components, the results in the present study are well aligned with previous cross-sectional studies demonstrating that parental education predicts childhood overweight and obesity [25]. In a recent cross-sectional study however, researchers found that household income was a stronger predictor of adverse health outcomes at age 17 years, than a deprivation index reflecting neighbourhood socioeconomic level [26]. Moreover, higher rates of overweight among children of immigrant descents have also been reported [27]. Taken together, evidence from cross-sectional associations indicates a multifactorial model where both individual-level and environmental socioeconomic factors are of importance. Previous research from the Nordic countries highlights the multifaceted role of socioeconomic status in childhood overweight and obesity. Parental education has shown consistent inverse associations with excess weight in both Denmark [28] and Norway [29]. Household income has similarly been linked to increased risk of unhealthy weight in Norwegian children [29]. In Finland, neighbourhood disadvantage has been associated with higher obesity risk [13, 30]. Moreover, immigrant background has been identified as an additional socioeconomic determinant in Denmark [28] and Norway [31]. Several studies have adjusted for other socioeconomic components [29, 31]. However, the relative importance of parental education, household income, and immigrant background, when evaluated within the same model, remains insufficiently understood. In the present study we observed that parental country of birth, educational level, and household income, were all associated with the diverging childhood BMI trend from 2008. Importantly, the upward BMI trend observed after 2008 was most pronounced in children with parents with low educational level, regardless of income or country of birth. The reasons for the adverse BMI trend in children with parents born outside Sweden, low educational level, or low income are not clear, but may be related to societal changes during these years. Inequalities in household disposable income and school segregation are reported to have increased in Sweden during the last 20 to 30 years [32, 33]. Furthermore, the emergence of an obesogenic environment, characterized by increased availability of energy-dense foods, limited access to affordable healthy options, and reduced opportunities for physical activity may have disproportionately affected families with fewer resources. It is conceivable that the diverging BMI trends may be attributable to differences in the uptake of preventive measures, inequalities in health, differential health behaviour, or access to healthcare. The findings in the present study suggest that the upward trajectory in childhood BMI has been reversed in certain parts of the population, whereas other parts exhibit an unfavourable trend. Taken together, these results point to insufficient societal efforts to curb unhealthy weight development in childhood.

Systematic reviews have demonstrated that in high-income countries, the association between socioeconomic factors and childhood obesity is bi-directional and complex [34]. Low parental socioeconomic status is associated with increased risk of obesity in their children, and childhood obesity has been shown to be causally associated with lower academic achievements [9, 35]. A Swedish study demonstrated higher risk of not completing 12 years of school in children with obesity independent of parental socioeconomic status [9]. These findings illustrate the overlap between low parental socioeconomic status, childhood obesity and subsequent low educational attainment in the children, increasing the risk of intergenerational health disparities.

The present study has several strengths, including the large population-based dataset with height and weight measured by trained school nurses and an extensive time span of six decades covering the entire obesity epidemic. In addition, using high-quality Swedish registers we have information on several socioeconomic components; parents’ country of birth, educational level, and income from the last two decades giving us the unique opportunity to evaluate differential BMI trends in socioeconomic strata. The limitations of the present study include the lack of data on parental BMI and lifestyle factors.

In conclusion, in the present study we demonstrate a significant increase in childhood BMI, overweight, and obesity from 1952 until 2001, followed by a pronounced decline. From 2008 we observe increasing social disparities where the trend for children with parents born in Sweden, with high educational level, and high income remains stable. In contrast, children to parents not born in Sweden, with low educational level, and low income displayed increasing BMI, overweight and obesity. The most pronounced upward trend in childhood BMI was seen in children with parents with low educational level, regardless of income or country of birth. Childhood obesity has an impact on health and the risk of disease, both in childhood and adulthood. The findings in the present study indicate increasing socioeconomic disparities in childhood health outcomes and motivates targeted interventions to ensure equity and a healthy start in life for all children. 

## Supplementary Information

Supplementary Material 1.

## Data Availability

Research data are not publicly available due to privacy and ethical restrictions. However, anonymized data that are minimally required to reproduce results can be made available from the corresponding author upon reasonable request, upon approval from the University of Gothenburg, if the data can be made available according to mandatory national law.
