# Supplementary material for: Six decades of trends in BMI, thinness, overweight, and obesity among 210,927 Swedish children in different socioeconomic contexts
Source: BMC Public Health. 2026 Sep 18;26:2627. doi: 10.1186/s12889-026-29521-4 (PMC13587539; doi:10.1186/s12889-026-29521-4)
Supplement: Supplementary file 1 [file 12889_2026_29521_MOESM1_ESM.docx]

**Supplementary material**

**Six decades of trends in BMI, thinness, overweight, and obesity among 210,927 Swedish children in different socioeconomic contexts**

Maria Bygdell, Klara Hemmed, Jari Martikainen, Carl Bonander, Lovisa Sjögren, Claes Ohlsson, Jenny M Kindblom

**Supplemental tables**

**Table S1.** Number of included children in schools in Gothenburg municipality, Sweden, by gender and for every year.

| **Year** | **Boys** | **Girls** | **Total** |
| --- | --- | --- | --- |
| 1952 | 1368 | 1295 | 2663 |
| 1953 | 1572 | 1690 | 3262 |
| 1954 | 2221 | 1912 | 4133 |
| 1955 | 2186 | 1872 | 4058 |
| 1956 | 2687 | 2471 | 5158 |
| 1957 | 2531 | 2412 | 4943 |
| 1958 | 2438 | 2749 | 5187 |
| 1959 | 2516 | 2307 | 4823 |
| 1960 | 2642 | 2546 | 5188 |
| 1961 | 2752 | 2801 | 5553 |
| 1962 | 2460 | 2795 | 5255 |
| 1963 | 3086 | 2948 | 6034 |
| 1964 | 3090 | 3025 | 6115 |
| 1965 | 3076 | 2881 | 5957 |
| 1966 | 2944 | 2727 | 5671 |
| 1967 | 2884 | 2837 | 5721 |
| 1968 | 3048 | 2931 | 5979 |
| 1969 | 3136 | 2923 | 6059 |
| 1970 | 3408 | 3139 | 6547 |
| 1971 | 3608 | 3301 | 6909 |
| 1972 | 3700 | 3273 | 6973 |
| 1973 | 3650 | 3240 | 6890 |
| 1974 | 3571 | 3172 | 6743 |
| 1975 | 2808 | 2603 | 5411 |
| 1978 | 595 | 1006 | 1601 |
| 1983 | 422 | 789 | 1211 |
| 1988 | 524 | 751 | 1275 |
| 1993 | 536 | 992 | 1528 |
| 1998 | 1435 | 1823 | 3258 |
| 1999 | 356 | 469 | 825 |
| 2000 | 1063 | 1023 | 2086 |
| 2001 | 1161 | 1194 | 2355 |
| 2002 | 1217 | 1204 | 2421 |
| 2003 | 1348 | 1308 | 2656 |
| 2004 | 1832 | 1830 | 3662 |
| 2005 | 2123 | 2090 | 4213 |
| 2006 | 2200 | 2185 | 4385 |
| 2008 | 2489 | 2312 | 4801 |
| 2009 | 2101 | 2154 | 4255 |
| 2010 | 2321 | 2282 | 4603 |
| 2011 | 2411 | 2193 | 4604 |
| 2012 | 2402 | 2305 | 4707 |
| 2013 | 2572 | 2446 | 5018 |
| 2014 | 2579 | 2505 | 5084 |
| 2015 | 2544 | 2360 | 4904 |
| 2016 | 2602 | 2480 | 5082 |
| 2017 | 2631 | 2530 | 5161 |
| **Total** | **106,846** | **104,081** | **210,927** |

**Table S2.** Mean childhood BMI and prevalence of BMI categories at the age of seven measured between 1952 and 2017 for 210,927 children in schools in Gothenburg municipality, Sweden, by gender and for every year.

| **Year** | **BMI**  Mean (SEM) | | **Thinness^1^**  n (%) | | **Normal weight^2^**  n (%) | | **Overweight^3^**  n (%) | | **Obesity^4^**  n (%) | |
| --- | --- | --- | --- | --- | --- | --- | --- | --- | --- | --- |
|  | **Boys** | **Girls** | **Boys** | **Girls** | **Boys** | **Girls** | **Boys** | **Girls** | **Boys** | **Girls** |
| 1952 | 15.52 ± 0.04 | 15.42 ± 0.04 | 127 (9.3%) | 132 (10.2%) | 1,171 (85.6%) | 1,077 (83.2%) | 64 (4.7%) | 71 (5.5%) | 6 (0.4%) | 15 (1.2%) |
| 1953 | 15.50 ± 0.03 | 15.45 ± 0.04 | 125 (8.0%) | 195 (11.5%) | 1,390 (88.4%) | 1,361 (80.5%) | 49 (3.1%) | 118 (7.0%) | 8 (0.5%) | 16 (0.9%) |
| 1954 | 15.52 ± 0.03 | 15.48 ± 0.03 | 190 (8.6%) | 169 (8.8%) | 1,939 (87.3%) | 1,617 (84.6%) | 80 (3.6%) | 107 (5.6%) | 12 (0.5%) | 19 (1.0%) |
| 1955 | 15.37 ± 0.03 | 15.30 ± 0.03 | 221 (10.1%) | 231 (12.3%) | 1,891 (86.5%) | 1,525 (81.5%) | 66 (3.0%) | 106 (5.7%) | 8 (0.4%) | 10 (0.5%) |
| 1956 | 15.55 ± 0.03 | 15.40 ± 0.03 | 244 (9.1%) | 266 (10.8%) | 2,319 (86.3%) | 2,058 (83.3%) | 104 (3.9%) | 134 (5.4%) | 20 (0.7%) | 13 (0.5%) |
| 1957 | 15.43 ± 0.02 | 15.44 ± 0.03 | 259 (10.2%) | 275 (11.4%) | 2,178 (86.1%) | 1,973 (81.8%) | 88 (3.5%) | 140 (5.8%) | 6 (0.2%) | 24 (1.0%) |
| 1958 | 15.46 ± 0.03 | 15.45 ± 0.03 | 249 (10.2%) | 301 (10.9%) | 2,084 (85.5%) | 2,235 (81.3%) | 84 (3.4%) | 184 (6.7%) | 21 (0.9%) | 29 (1.1%) |
| 1959 | 15.41 ± 0.03 | 15.37 ± 0.03 | 299 (11.9%) | 282 (12.2%) | 2,101 (83.5%) | 1,869 (81.0%) | 102 (4.1%) | 130 (5.6%) | 14 (0.6%) | 26 (1.1%) |
| 1960 | 15.45 ± 0.03 | 15.51 ± 0.03 | 273 (10.3%) | 241 (9.5%) | 2,249 (85.1%) | 2,110 (82.9%) | 107 (4.0%) | 164 (6.4%) | 13 (0.5%) | 31 (1.2%) |
| 1961 | 15.57 ± 0.03 | 15.52 ± 0.03 | 244 (8.9%) | 308 (11.0%) | 2,358 (85.7%) | 2,237 (79.9%) | 128 (4.7%) | 230 (8.2%) | 22 (0.8%) | 26 (0.9%) |
| 1962 | 15.63 ± 0.03 | 15.57 ± 0.03 | 216 (8.8%) | 301 (10.8%) | 2,086 (84.8%) | 2,238 (80.1%) | 140 (5.7%) | 222 (7.9%) | 18 (0.7%) | 34 (1.2%) |
| 1963 | 15.55 ± 0.03 | 15.49 ± 0.03 | 310 (10.0%) | 345 (11.7%) | 2,627 (85.1%) | 2,357 (80.0%) | 118 (3.8%) | 214 (7.3%) | 31 (1.0%) | 32 (1.1%) |
| 1964 | 15.57 ± 0.03 | 15.53 ± 0.03 | 282 (9.1%) | 300 (9.9%) | 2,650 (85.8%) | 2,449 (81.0%) | 131 (4.2%) | 246 (8.1%) | 27 (0.9%) | 30 (1.0%) |
| 1965 | 15.53 ± 0.03 | 15.47 ± 0.03 | 307 (10.0%) | 338 (11.7%) | 2,597 (84.4%) | 2,297 (79.7%) | 150 (4.9%) | 208 (7.2%) | 22 (0.7%) | 38 (1.3%) |
| 1966 | 15.60 ± 0.03 | 15.54 ± 0.03 | 261 (8.9%) | 294 (10.8%) | 2,517 (85.5%) | 2,175 (79.8%) | 139 (4.7%) | 216 (7.9%) | 27 (0.9%) | 42 (1.5%) |
| 1967 | 15.55 ± 0.03 | 15.54 ± 0.03 | 288 (10.0%) | 345 (12.2%) | 2,441 (84.6%) | 2,224 (78.4%) | 134 (4.6%) | 225 (7.9%) | 21 (0.7%) | 43 (1.5%) |
| 1968 | 15.53 ± 0.03 | 15.49 ± 0.03 | 296 (9.7%) | 336 (11.5%) | 2,592 (85.0%) | 2,310 (78.8%) | 141 (4.6%) | 246 (8.4%) | 19 (0.6%) | 39 (1.3%) |
| 1969 | 15.46 ± 0.02 | 15.48 ± 0.03 | 309 (9.9%) | 360 (12.3%) | 2,683 (85.6%) | 2,316 (79.2%) | 124 (4.0%) | 204 (7.0%) | 20 (0.6%) | 43 (1.5%) |
| 1970 | 15.64 ± 0.02 | 15.66 ± 0.03 | 260 (7.6%) | 281 (9.0%) | 2,934 (86.1%) | 2,537 (80.8%) | 185 (5.4%) | 275 (8.8%) | 29 (0.9%) | 46 (1.5%) |
| 1971 | 15.65 ± 0.02 | 15.58 ± 0.03 | 297 (8.2%) | 347 (10.5%) | 3,071 (85.1%) | 2,631 (79.7%) | 196 (5.4%) | 278 (8.4%) | 44 (1.2%) | 45 (1.4%) |
| 1972 | 15.61 ± 0.02 | 15.63 ± 0.03 | 302 (8.2%) | 297 (9.1%) | 3,184 (86.1%) | 2,673 (81.7%) | 184 (5.0%) | 252 (7.7%) | 30 (0.8%) | 51 (1.6%) |
| 1973 | 15.63 ± 0.02 | 15.61 ± 0.03 | 297 (8.1%) | 303 (9.4%) | 3,129 (85.7%) | 2,608 (80.5%) | 199 (5.5%) | 276 (8.5%) | 25 (0.7%) | 53 (1.6%) |
| 1974 | 15.64 ± 0.02 | 15.65 ± 0.03 | 317 (8.9%) | 311 (9.8%) | 3,066 (85.9%) | 2,560 (80.7%) | 165 (4.6%) | 246 (7.8%) | 23 (0.6%) | 55 (1.7%) |
| 1975 | 15.66 ± 0.03 | 15.62 ± 0.03 | 223 (7.9%) | 267 (10.3%) | 2,419 (86.1%) | 2,085 (80.1%) | 144 (5.1%) | 217 (8.3%) | 22 (0.8%) | 34 (1.3%) |
| 1978 | 15.84 ± 0.05 | 15.84 ± 0.05 | 27 (4.5%) | 65 (6.5%) | 532 (89.4%) | 818 (81.3%) | 33 (5.5%) | 106 (10.5%) | 3 (0.5%) | 17 (1.7%) |
| 1983 | 15.95 ± 0.08 | 15.86 ± 0.06 | 30 (7.1%) | 71 (9.0%) | 351 (83.2%) | 610 (77.3%) | 35 (8.3%) | 89 (11.3%) | 6 (1.4%) | 19 (2.4%) |
| 1988 | 15.95 ± 0.08 | 15.92 ± 0.07 | 42 (8.0%) | 59 (7.9%) | 426 (81.3%) | 581 (77.4%) | 43 (8.2%) | 94 (12.5%) | 13 (2.5%) | 17 (2.3%) |
| 1993 | 16.17 ± 0.08 | 16.27 ± 0.06 | 32 (6.0%) | 55 (5.5%) | 430 (80.2%) | 741 (74.7%) | 56 (10.4%) | 149 (15.0%) | 18 (3.4%) | 47 (4.7%) |
| 1998 | 16.37 ± 0.05 | 16.24 ± 0.05 | 82 (5.7%) | 145 (8.0%) | 1,101 (76.7%) | 1,289 (70.7%) | 176 (12.3%) | 305 (16.7%) | 76 (5.3%) | 84 (4.6%) |
| 1999 | 16.20 ± 0.10 | 16.47 ± 0.11 | 25 (7.0%) | 33 (7.0%) | 280 (78.7%) | 333 (71.0%) | 37 (10.4%) | 69 (14.7%) | 14 (3.9%) | 34 (7.2%) |
| 2000 | 16.26 ± 0.06 | 16.43 ± 0.08 | 79 (7.4%) | 79 (7.7%) | 813 (76.5%) | 694 (67.8%) | 124 (11.7%) | 178 (17.4%) | 47 (4.4%) | 72 (7.0%) |
| 2001 | 16.41 ± 0.06 | 16.31 ± 0.07 | 77 (6.6%) | 91 (7.6%) | 873 (75.2%) | 858 (71.9%) | 154 (13.3%) | 170 (14.2%) | 57 (4.9%) | 75 (6.3%) |
| 2002 | 16.33 ± 0.06 | 16.23 ± 0.06 | 87 (7.1%) | 77 (6.4%) | 918 (75.4%) | 901 (74.8%) | 148 (12.2%) | 172 (14.3%) | 64 (5.3%) | 54 (4.5%) |
| 2003 | 16.32 ± 0.06 | 16.42 ± 0.06 | 100 (7.4%) | 80 (6.1%) | 1,001 (74.3%) | 934 (71.4%) | 188 (13.9%) | 210 (16.1%) | 59 (4.4%) | 84 (6.4%) |
| 2004 | 16.16 ± 0.05 | 16.16 ± 0.05 | 144 (7.9%) | 136 (7.4%) | 1,414 (77.2%) | 1,357 (74.2%) | 212 (11.6%) | 253 (13.8%) | 62 (3.4%) | 84 (4.6%) |
| 2005 | 16.08 ± 0.04 | 16.10 ± 0.04 | 169 (8.0%) | 163 (7.8%) | 1,687 (79.5%) | 1,583 (75.7%) | 202 (9.5%) | 257 (12.3%) | 65 (3.1%) | 87 (4.2%) |
| 2006 | 16.08 ± 0.04 | 16.09 ± 0.04 | 146 (6.6%) | 178 (8.1%) | 1,756 (79.8%) | 1,620 (74.1%) | 231 (10.5%) | 292 (13.4%) | 67 (3.0%) | 95 (4.3%) |
| 2008 | 16.09 ± 0.04 | 16.02 ± 0.04 | 201 (8.1%) | 178 (7.7%) | 1,940 (77.9%) | 1,760 (76.1%) | 268 (10.8%) | 290 (12.5%) | 80 (3.2%) | 84 (3.6%) |
| 2009 | 16.02 ± 0.04 | 16.04 ± 0.04 | 174 (8.3%) | 154 (7.1%) | 1,658 (78.9%) | 1,652 (76.7%) | 203 (9.7%) | 276 (12.8%) | 66 (3.1%) | 72 (3.3%) |
| 2010 | 16.07 ± 0.04 | 16.06 ± 0.04 | 192 (8.3%) | 166 (7.3%) | 1,819 (78.4%) | 1,747 (76.6%) | 234 (10.1%) | 278 (12.2%) | 76 (3.3%) | 91 (4.0%) |
| 2011 | 16.08 ± 0.04 | 16.13 ± 0.04 | 181 (7.5%) | 174 (7.9%) | 1,901 (78.8%) | 1,633 (74.5%) | 245 (10.2%) | 294 (13.4%) | 84 (3.5%) | 92 (4.2%) |
| 2012 | 16.05 ± 0.04 | 16.10 ± 0.04 | 184 (7.7%) | 181 (7.9%) | 1,915 (79.7%) | 1,727 (74.9%) | 230 (9.6%) | 298 (12.9%) | 73 (3.0%) | 99 (4.3%) |
| 2013 | 16.03 ± 0.04 | 15.99 ± 0.04 | 206 (8.0%) | 193 (7.9%) | 2,021 (78.6%) | 1,866 (76.3%) | 259 (10.1%) | 293 (12.0%) | 86 (3.3%) | 94 (3.8%) |
| 2014 | 16.00 ± 0.04 | 16.07 ± 0.04 | 237 (9.2%) | 195 (7.8%) | 2,017 (78.2%) | 1,892 (75.5%) | 244 (9.5%) | 315 (12.6%) | 81 (3.1%) | 103 (4.1%) |
| 2015 | 16.12 ± 0.04 | 16.13 ± 0.04 | 239 (9.4%) | 193 (8.2%) | 1,925 (75.7%) | 1,724 (73.1%) | 271 (10.7%) | 346 (14.7%) | 109 (4.3%) | 97 (4.1%) |
| 2016 | 16.06 ± 0.04 | 16.08 ± 0.04 | 242 (9.3%) | 212 (8.5%) | 1,970 (75.7%) | 1,835 (74.0%) | 286 (11.0%) | 340 (13.7%) | 104 (4.0%) | 93 (3.8%) |
| 2017 | 16.12 ± 0.04 | 16.08 ± 0.04 | 228 (8.7%) | 195 (7.7%) | 1,990 (75.6%) | 1,872 (74.0%) | 312 (11.9%) | 364 (14.4%) | 101 (3.8%) | 99 (3.9%) |
| **Total** |  |  | 9,320 (8.7%) | 9,898 (9.5%) | 88,414 (82.7%) | 81,549 (78.4%) | 7,213 (6.8%) | 10,147 (9.7%) | 1,899 (1.8%) | 2,487 (2.4%) |

^1^Thinness for boys <14.00 kg/m^2^ and for girls <13.83 kg/m^2^,

^2^Normal Weight for boys ≥14.00<17.88 kg/m^2^ and for girls ≥13.83<17.69 kg/m^2^,

^3^Overweight for boys ≥17.88<20.59 kg/m^2^ and for girls ≥17.69<20.39 kg/m^2^,

^4^Obesity for boys ≥20.59 kg/m^2^ and for girls ≥20.39 kg/m^2^ according to International Obesity Task Force (1)

**Table S3.** Correlations between the parental socioeconomic variables showed by Pearson’s correlation coefficient. The study population included 65,556 children in schools in Gothenburg municipality, Sweden

|  | **Parental country of birth** | **Parental educational level** | **Parental income** |
| --- | --- | --- | --- |
| **Parental country of birth** | 1 | 0.23 | 0.43 |
| **Parental educational level** | 0.23 | 1 | 0.33 |
| **Parental income** | 0.43 | 0.33 | 1 |

**Table S4.** Number of children with BMI at the age of seven between 2002 and 2017 for 65,556 children in schools in Gothenburg municipality, Sweden, by gender and parental socioeconomic strata.

| **Year** | **Parental country of birth^1^** | | | | **Parental educational level^1^** | | | | **Parental income^1^** | | | | | |  |
| --- | --- | --- | --- | --- | --- | --- | --- | --- | --- | --- | --- | --- | --- | --- | --- |
|  | **Sweden**  n (%) | | **Not Sweden**  n (%) | | **High**  n (%) | | **Low**  n (%) | | | **High**  n (%) | | | **Low**  n (%) | | |
|  | **Boys** | **Girls** | **Boys** | **Girls** | **Boys** | **Girls** | **Boys** | **Girls** | **Boys** | | **Girls** | **Boys** | | **Girls** |  |
| 2002 | 702 (57.7%) | 711 (59.1%) | 515 (42.3%) | 493 (40.9%) | 612 (50.3%) | 591 (49.1%) | 596 (49.0%) | 589 (48.9%) | 618 (50.8%) | | 584 (48.5%) | 595 (48.9%) | | 609 (50.6%) |  |
| 2003 | 806 (59.8%) | 817 (62.5%) | 542 (40.2%) | 491 (37.5%) | 701 (52.0%) | 653 (49.9%) | 634 (47.0%) | 631 (48.2%) | 668 (49.6%) | | 648 (49.5%) | 673 (49.9%) | | 645 (49.3%) |  |
| 2004 | 1,143 (62.4%) | 1,071 (58.5%) | 689 (37.6%) | 759 (41.5%) | 996 (54.4%) | 934 (51.0%) | 819 (44.7%) | 872 (47.7%) | 919 (50.2%) | | 898 (49.1%) | 903 (49.3%) | | 917 (50.1%) |  |
| 2005 | 1,265 (59.6%) | 1,229 (58.8%) | 858 (40.4%) | 861 (41.2%) | 1,130 (53.2%) | 1,106 (52.9%) | 971 (45.7%) | 956 (45.7%) | 1,059 (49.9%) | | 1,034 (49.5%) | 1,057 (49.8%) | | 1,039 (49.7%) |  |
| 2006 | 1,298 (59.0%) | 1,274 (58.3%) | 902 (41.0%) | 911 (41.7%) | 1,154 (52.5%) | 1,170 (53.5%) | 1,019 (46.3%) | 992 (45.4%) | 1,106 (50.3%) | | 1,074 (49.2%) | 1,084 (49.3%) | | 1,103 (50.5%) |  |
| 2008 | 1,449 (58.2%) | 1,362 (58.9%) | 1,040 (41.8%) | 950 (41.1%) | 1,413 (56.8%) | 1,284 (55.5%) | 1,054 (42.3%) | 1,001 (43.3%) | 1,238 (49.7%) | | 1,150 (49.7%) | 1,241 (49.9%) | | 1,154 (49.9%) |  |
| 2009 | 1,140 (54.3%) | 1,187 (55.1%) | 961 (45.7%) | 967 (44.9%) | 1,229 (58.5%) | 1,276 (59.2%) | 844 (40.2%) | 854 (39.6%) | 1,056 (50.3%) | | 1,058 (49.1%) | 1,031 (49.1%) | | 1,086 (50.4%) |  |
| 2010 | 1,285 (55.4%) | 1,235 (54.1%) | 1,036 (44.6%) | 1,047 (45.9%) | 1,384 (59.6%) | 1,359 (59.6%) | 907 (39.1%) | 896 (39.3%) | 1,153 (49.7%) | | 1,130 (49.5%) | 1,154 (49.7%) | | 1,138 (49.9%) |  |
| 2011 | 1,278 (53.0%) | 1,205 (54.9%) | 1,133 (47.0%) | 988 (45.1%) | 1,471 (61.0%) | 1,346 (61.4%) | 918 (38.1%) | 820 (37.4%) | 1,177 (48.8%) | | 1,109 (50.6%) | 1,220 (50.6%) | | 1,070 (48.8%) |  |
| 2012 | 1,297 (54.0%) | 1,201 (52.1%) | 1,105 (46.0%) | 1,104 (47.9%) | 1,463 (60.9%) | 1,397 (60.6%) | 909 (37.8%) | 873 (37.9%) | 1,198 (49.9%) | | 1,137 (49.3%) | 1,186 (49.4%) | | 1,150 (49.9%) |  |
| 2013 | 1,344 (52.3%) | 1,320 (54.0%) | 1,228 (47.7%) | 1,126 (46.0%) | 1,573 (61.2%) | 1,489 (60.9%) | 964 (37.5%) | 918 (37.5%) | 1,272 (49.5%) | | 1,216 (49.7%) | 1,277 (49.7%) | | 1,211 (49.5%) |  |
| 2014 | 1,360 (52.7%) | 1,301 (51.9%) | 1,219 (47.3%) | 1,204 (48.1%) | 1,665 (64.6%) | 1,556 (62.1%) | 879 (34.1%) | 910 (36.3%) | 1,323 (51.3%) | | 1,199 (47.9%) | 1,240 (48.1%) | | 1,283 (51.2%) |  |
| 2015 | 1,329 (52.2%) | 1,180 (50.0%) | 1,215 (47.8%) | 1,180 (50.0%) | 1,618 (63.6%) | 1,482 (62.8%) | 892 (35.1%) | 845 (35.8%) | 1,252 (49.2%) | | 1,176 (49.8%) | 1,269 (49.9%) | | 1,159 (49.1%) |  |
| 2016 | 1,322 (50.8%) | 1,304 (52.6%) | 1,280 (49.2%) | 1,176 (47.4%) | 1,675 (64.4%) | 1,593 (64.2%) | 890 (34.2%) | 856 (34.5%) | 1,286 (49.4%) | | 1,233 (49.7%) | 1,292 (49.7%) | | 1,231 (49.6%) |  |
| 2017 | 1,317 (50.1%) | 1,287 (50.9%) | 1,314 (49.9%) | 1,243 (49.1%) | 1,691 (64.3%) | 1,629 (64.4%) | 918 (34.9%) | 876 (34.6%) | 1,331 (50.6%) | | 1,242 (49.1%) | 1,294 (49.2%) | | 1,281 (50.6%) |  |
| **Total** | 18,335 (54.9%) | 17,684 (54.9%) | 15,037 (45.1%) | 14,500 (45.1%) | 19,775 (59.3%) | 18,865 (58.6%) | 13,214 (39.6%) | 12,889 (40.0%) | 16,656 (49.9%) | | 15,888 (49.4%) | 16,516 (49.5%) | | 16,076 (50.0%) |  |

^1^Number of children with missing data in parental country of birth (n=0), parental educational level (n=813), and parental income (n=420).

**Supplemental figures**

**Figure S1.** Flow chart of the inclusion process of the study population.

**Figure S2.** Mean (dots) and standard error (lines) of childhood BMI at the age of seven measured between 1952 and 2017 for 210,927 children in schools in Gothenburg municipality, Sweden, by gender and for every year.

**Figure S3.** Childhood BMI at the age of seven measured between 1952 and 2017 for 210,927 children in schools in Gothenburg municipality, Sweden, for the 5^th^, 25^th^, 50^th^, 75^th^, 85^th^, 95^th^, and 99^th^ percentiles in each year and splines between the years obtained from quantile regression. Dotted lines indicate thinness, overweight, and obesity categorized using the definitions proposed by the International Obesity Task Force (1).

**Figure S4.** Trend in BMI categories at the age of seven between 1952 and 2017 for 210,927 children in schools in Gothenburg municipality, Sweden. Proportions according to BMI categories each year (dots) and raw data fitted with splines and their 95% confidence intervals. Cut-offs for the BMI groups were derived from the International Obesity Task Force (1). Note that the y-axis has another dimension for the plot of normal weight compared to the others.

**Figure S5.** Cross-sectional analysis of the associations between different socioeconomic variables (parental country of birth, parental educational level, and parental income) and childhood BMI at the age of seven between 2002 and 2017 for 65,556 children in schools in Gothenburg municipality, Sweden. Beta and 95% confidence intervals from linear regression including all three socioeconomic components as well as gender, parental age at childbirth, and year (linear and non-linear) in the same model.

Parental country of birth (reference: Sweden), educational level (reference: high), and income (reference: high).

**Figure S6.** The trend in BMI at the age of seven between 2002 and 2017 for 63,888 children in schools in Gothenburg municipality, Sweden, by parental country of birth. Regression lines and their 95% confidence intervals from a piecewise linear regression model with one breakpoint and including gender, parents’ age at childbirth, and the socioeconomic variables not stratified on.

**Figure S7.** The odds for overweight and obesity at the age of seven between 2002 and 2017 for 63,888 children in schools in Gothenburg municipality, Sweden, by parental country of birth. Odds ratio and their 95% confidence intervals per five years from logistic regression models in the two phases and including gender, parents’ age at childbirth, and the socioeconomic variables not stratified on. Overweight and obesity compared to not overweight and not obesity, respectively.

**References**

1.	Cole TJ, Lobstein T. Extended international (IOTF) body mass index cut-offs for thinness, overweight and obesity. Pediatr Obes. 2012 Aug;7(4):284-94.
